# The Medical Profession and Legislation against Contraceptives

**Published:** 1917-05-12

**Authors:** 


### THE MEDICAL PROFESSION AND LEGISLATION AGAINST CONTRACEPTIVES.

We learn from our contemporary the *South African Medical Record* that to a Government Bill now before the Senate, and dealing with medical and pharmaceutical questions, one of the Senators has given notice of an amendment proposing to prohibit, except on the order of a medical practitioner, the sale of mechanical contrivances intended for the prevention of conception. The proposal, it appears, has engaged the favourable attention of the Cape Medical Council, and the editor of the *Medical Record* gives it his warm support. Or, more exactly, he urges that the prohibition should be an absolute one, though if this ideal is beyond reach he will accept the dispensing power conferred on the profession. Sooner or later the question is likely to be raised in this country, where, as well as at the Cape, the articles in question have a large and probably an increasing sale. The subject is one which has many aspects and is hardly likely to be regarded merely as a medical problem. At the same time, the medical profession will expect to be heard on the matter, and more particularly if, as is now suggested in South Africa, there should be imposed on the practitioner the responsibility of judging in individual cases whether the methods here in question should or should not be sanctioned. It is not difficult to imagine an effort of the Legislature directed to the complete suppression of these devices. That they are employed in an immoral fashion both by married and by unmarried persons is beyond dispute, and the declining birth-rate carries its own message. Hence the State might decree that their employment is prejudicial to the national strength and safety and might seek to suppress their sale. In this respect the issue is one of public policy and must be decided by public opinion. As citizens members of the medical profession would be entitled to their individual opinions on the matter, and many of them without question would feel entitled to take part in the education of their fellows. What we wish to say at the moment is that while total prohibition may be defended, we should oppose any legislation which would compel the practitioner to decide for or against these methods as medical proposals for individual patients. Prohibition may stand or fall on its own claims. But a dispensing power inflicted on the profession as now suggested at the Cape would, it seems to us, create a position altogether intolerable.

The broad reason for our objection is that there is no common agreement in the profession on the circumstances which may be held to justify the prevention of conception. What one man may quite honestly regard as sufficient another will dismiss as wholly inadequate. There are, too, social students of high standing who hold strongly that limitation in the size of families is both an advantage to the State and a moral duty; and some of these are medical practitioners. We are not pretending just now either to condemn or to endorse this thesis. But we do say that so long as such sharp differences of opinion exist it would be an intolerable burden for a medical man to be obliged to face the issue on the invitation of an individual patient. The more confidently it is affirmed that these appliances have a wide use the more severe obviously would be the pressure to which the practitioner would be exposed. Of course, there are methods of preventing conception which, however deserving of moral censure, no legislation could possibly touch. These are not now within the discussion. What is at issue is the sale of certain mechanical contrivances specially adapted for a restrictive purpose. The State may conclude that such sale is contrary to public policy, and may, if it seems good, legislate to suppress it. Then the course of the medical practitioner is clear; with illegal proceedings he can have no association. But he ought not to be compelled to act as an arbitrator in a delicate question of morals and where no clear standard of duty has yet been defined. Nearly everyone allows that the methods now under consideration are often practised from unworthy motives. If the practice is to the nation's prejudice let the Legislature suppress it so far as this is possible. But when this is done the medical profession may reasonably be excused from an attempt to pick out exceptions in a scheme of sanctions which needs must vary with the individual judgment.

